# Association between the triglyceride glucose index and length of hospital stay in patients with heart failure and type 2 diabetes in the intensive care unit: a retrospective cohort study

**DOI:** 10.3389/fendo.2024.1354614

**Published:** 2024-05-10

**Authors:** Kai Zhang, Yu Han, Yu Xuan Gao, Fang Ming Gu, Tianyi Cai, Zhao Xuan Gu, Zhao Jia Yu, Gao Min, Ya Fang Gao, Rui Hu, Mao Xun Huang

**Affiliations:** ^1^ Cardiovascular Surgery Department, The Second Hospital of Jilin University, Changchun, China; ^2^ Department of Ophthalmology, First Hospital of Jilin University, Changchun, China; ^3^ Department of Ophthalmology, Second Hospital of Jilin University, Changchun, China; ^4^ Department of Cancer Center, The First Hospital of Jilin University, Changchun, China

**Keywords:** triglyceride-glucose index, heart failure, diabetes, generalized additive model, length of hospital stay

## Abstract

**Background:**

The coexistence of heart failure and diabetes is prevalent, particularly in Intensive Care Units (ICU). However, the relationship between the triglyceride-glucose (TyG) index, heart failure, diabetes, and the length of hospital stay (LHS) in patients with cerebrovascular disease in the ICU remains uncertain. This study aims to investigate the association between the TyG index and LHS in patients with heart failure and diabetes.

**Methods:**

This retrospective study utilized the Medical Information Mart for Intensive Care (MIMIC)-IV database to analyze patients with diabetes and heart failure. Participants were categorized into quartiles based on the TyG index, and the primary outcome was LHS. The association between the TyG index at ICU admission and LHS was examined through multivariable logistic regression models, restricted cubic spline regression, and subgroup analysis.

**Results:**

The study included 635 patients with concurrent diabetes and heart failure. The fully adjusted model demonstrated a positive association between the TyG index and LHS. As a tertile variable (Q2 and Q3 vs Q1), the beta (β) values were 0.88 and 2.04, with a 95% confidence interval (95%CI) of -0.68 to 2.44 and 0.33 to 3.74, respectively. As a continuous variable, per 1 unit increment, the β (95% CI) was 1.13 (0.18 to 2.08). The TyG index’s relationship with LHS showed linearity (non-linear p = 0.751). Stratified analyses further confirmed the robustness of this correlation.

**Conclusion:**

The TyG index exhibited a linearly positive association with the LHS in patients with both heart failure and diabetes. Nevertheless, prospective, randomized, controlled studies are imperative to substantiate and validate the findings presented in this investigation.

## Introduction

Diabetes mellitus (DM) encompasses metabolic disorders marked by persistent hyperglycemia and compromised metabolism of carbohydrates, lipids, and proteins due to deficiencies in insulin secretion, action, or both (1). Projections from the International Diabetes Federation anticipate a rise to 578 million adults with diabetes by 2030 and 700 million by 2045 (2, 3). Notably, over 90% of global diabetes cases fall under type 2 diabetes mellitus (T2D) (4), which, rather than manifesting in isolation, manifests as a component of a multifaceted metabolic‐cardiovascular syndrome (5). Epidemiological investigations have firmly established a significant correlation between heart failure and DM (6–8). Specifically, T2D acts as a cardiovascular risk factor, elevating the likelihood of heart failure and intensifying morbidity and mortality risks in affected individuals (9). Heart failure surpasses myocardial infarction or stroke as the most prevalent cardiovascular complication of diabetes (10). Previous studies suggest that individuals with T2D represent a distinct population with heightened cardiovascular risk, necessitating tailored prevention programs (11). Therefore, assessing the progression in patients with both heart failure and T2D is imperative for optimizing personalized treatment strategies.

The Triglyceride-Glucose (TyG) index, derived from fasting triglyceride and fasting blood glucose, emerges as a novel surrogate measure for insulin resistance, exhibiting superior diagnostic and predictive capabilities for diabetes compared to blood glucose alone (12, 13). Several studies associate the TyG index with atherosclerosis, metabolic syndrome, and T2D (14–16), while others explore its relevance in heart failure and severe illnesses (17). Recognizing the shared pathophysiological mechanisms between diabetes and heart failure underscores the significance of managing their synergistic effects (9, 10). Nevertheless, the linkage between the TyG index and the length of hospital stay (LHS) in patients with both T2D and heart failure (HF) remains unexplored.

Previous research underscores the importance of LHS as an indicator for assessing postoperative functional recovery, correlating with postoperative complications and resource utilization (18, 19). This study endeavors to examine the association between the TyG index and the risk of in-hospital events and LHS in patients with combined T2D and HF admitted to the intensive care unit (ICU). This exploration may assist in identifying high-risk patients, warranting closer monitoring or early intervention.

## Method

### Data source

This retrospective observational study utilized data from the publicly accessible Medical Information Mart for Intensive Care IV (MIMIC-IV) database, which contains comprehensive clinical information from patients treated at Beth Israel Deaconess Hospital in Boston, Massachusetts, USA (20, 21). The dataset encompasses demographics, vital signs, test findings, and diagnoses coded with International Classification of Diseases and Ninth Revision (ICD-9) and International Classification of Diseases and Tenth Revision (ICD-10) (22). Access to the MIMIC-IV database was granted to the primary author, Kai Zhang, subsequent to successful completion of the ‘Protecting Human Research Participants’ examination (ID: 11639604) administered by the National Institutes of Health (NIH). The data within MIMIC-IV had been previously deidentified, obviating the need for a waiver for informed consent (23). Given the public and anonymized nature of the MIMIC database, ethical committee approval was exempted (24). Reporting of this cross-sectional study adhered to the Strengthening the Reporting of Observational Studies in Epidemiology (STROBE) statement.

### Study population

**Figure 1:**
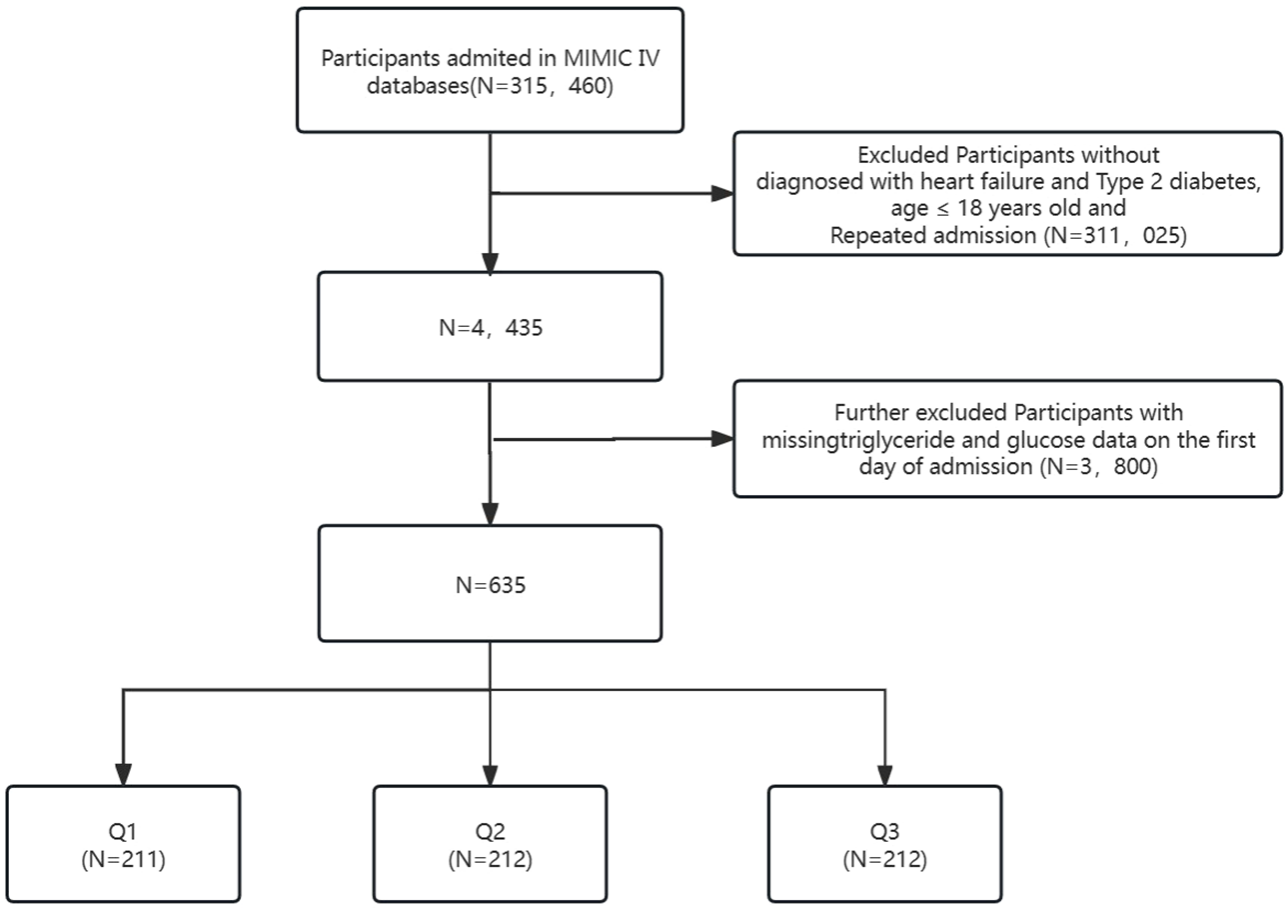
Flowchart detailing the selection process for patients included in this analysis.

This investigation encompassed individuals diagnosed with HF and T2D identified through ICD-9 and ICD-10 codes (https://icd.who.int/browse10/2019/en). **Supplementary Material** provides specific diagnostic codes. Exclusions comprised patients under 18 years old at admission, those without HF and T2D diagnoses, and those with repeated admissions, resulting in a total exclusion of 311,025 individuals from an initial pool of 315,460 ICU-admitted patients in MIMIC-IV. An additional 3,800 individuals lacking triglyceride (TG) and glucose data on the first admission day were excluded. The final study cohort comprised 635 patients categorized into three groups based on the Terciles of the TyG index on the first day of ICU stay (see **Figure 1**).

### Data collection

The study utilized PostgresSQL (version 13.7.2) and Navicate Premium (version 16) for information extraction through a running Structured Query Language (SQL) (25, 26). Data extraction from the MIMIC-IV database encompassed demographics, lab results, hourly vital signs, comorbidities, medications, surgical procedures, and ICU stay specifics, facilitated by pgAdmin PostgreSQL tools (version 1.22.1).

The extraction of potential variables could be divided into six main groups: (1) demographic variables (gender, age, marital status, body mass index. (2) Vital signs (heart rate, systolic pressure, diastolic pressure, respiratory rate, Pulse Oxygen Saturation (SpO2), temperature). (3) Comorbidities (cirrhosis, hyperlipidemia, chronic kidney disease (CKD), chronic obstructive pulmonary disease (COPD), hypertension). (4) Medication(dopamine, epinephrine, dobutamine) (5) Laboratory indicators(white blood cell count(WBC), red blood cell count(RBC), platelet count, hemoglobin, albumin, sodium, potassium, calcium total, chloride, partial pressure of carbon dioxide(pCO2), Oxygen partial pressure(pO2), free calcium, prothrombin time(PT), activated partial thromboplastin time(APTT), high density lipoprotein (HDL), low density lipoprotein(LDL), glutamic-pyruvic transaminase(ALT), glutamic oxaloacetic transaminase(AST), urea nitrogen, creatinine, lactic dehydrogenase(LD), creatine kinase(CK), Creatine kinase isoenzymes(CKMB0) (6)severity of illness scores at admission, including the Acute Physiology Score III (APSIII), the simplified Acute Physiology Score II (SAPS-II), and the Sepsis-related Organ Failure Assessment score (SOFA) (27, 28). Follow- up began on the date of admission and ended on the date of death. Demographic characteristics and vital signs within the first 24 hours of admission were captured, with the initial measurements at admission serving as laboratory examination indicators.

### Expose and outcome measures

Baseline TyG index was calculated based on fasting TGs and FBG values obtained at admission as previously reported: ln [fasting TGs (mg/dL) * FBG (mg/dL)/2] (29). The participants were divided into three groups according to the Tierce level of the TyG index. The primary outcome indicator was the patient’s LHS.

### Statistical analyses

The data were initially categorized into continuous and categorical variables. Continuous variables were expressed as mean (SD) or median (IQR) and compared using appropriate statistical tests, such as the student t-test or nonparametric tests. Categorical variables were presented as frequencies and percentages (%) and compared using the Pearson chi-square test or Fisher’s exact test. Baseline characteristics were grouped according to Tertiles of the TyG index.

To assess the relationship between the TyG index and LHS, multivariable logistic regression analyses were conducted. The models progressively adjusted for covariates: Model 1 included no adjusted covariates. Model 2 included demographic variables (gender, age, marital status, body mass index). Model 3 added vital signs and comorbidities. Model 4 further included medications and laboratory indicators. Model 5 additionally incorporated severity of illness scores at admission. Trend tests utilized logistic regression with the TyG index categorized into three groups. Variables with a variance inflation factor exceeding 5 were excluded to prevent multicollinearity.

Potential nonlinear correlations between TyG index levels and outcomes were explored using restricted cubic splines. Stratified regression models and likelihood ratio tests identified modifications and interactions in subgroups based on factors such as age, gender, BMI, hyperlipidemia, chronic kidney disease, and hypertension. Missing values for laboratory indicators in the MIMIC-IV database were addressed by calculating the percentage of missing values for each continuous variable. Imputation was performed using a random forest-based method for variables with less than 20% missing values, while variables with over 20% missing values were categorized. Non-normally distributed continuous variables were analyzed after conversion into categorical variables based on the normal reference range from the MIMIC-IV database.

All statistical analyses were conducted using R, version 4.1.1, and Free Statistics software, version 1.7 (30). A significance level of P < 0.05 (two-sided) was considered statistically significant. Reporting adhered to the Strengthening the Reporting of Observational Studies in Epidemiology (STROBE) statement for this cross-sectional study.

## Results

### Baseline characteristics of selected participants

**Table 1** presents the baseline characteristics of 635 patients diagnosed with both diabetes and congestive heart failure (CHF). Stratified into tertiles based on the TyG index (Q1 ≤ 8.984, Q2 8.984-9.625, Q3 ≥ 9.625), the entire patient cohort had a mean age of 71.0 ± 12.3 years. High TyG index individuals, regardless of gender, exhibited characteristics such as youthfulness, elevated BMI, increased heart and respiratory rates, reduced Spo2, heightened WBC, lowered Chlorine, diminished HDL, elevated LDL, increased LD, higher APSIII and SOFA scores, and extended LHS compared to their low TyG index counterparts.

**Table 1:** Characteristics of the study population (N = 635).

Variables	Total (n = 635)	Q1^b^ (n = 211)	Q2^b^ (n = 212)	Q3^b^ (n = 212)	P value^a^
Demographics					
Age, Mean ± SD	71.0 ± 12.3	72.8 ± 12.1	72.6 ± 11.2	67.6 ± 12.7	< 0.001
Gender, n (%)					0.524
Female	250 (39.4)	79 (37.4)	90 (42.5)	81 (38.2)	
Male	385 (60.6)	132 (62.6)	122 (57.5)	131 (61.8)	
Body mass index, Mean ± SD	32.6 ± 9.5	30.8 ± 9.4	33.1 ± 9.3	34.1 ± 9.7	0.001
Marital status, n (%)					0.374
DIVORCED	57 ( 9.0)	25 (11.8)	18 (8.5)	14 (6.6)	
MARRIED	340 (53.5)	111 (52.6)	110 (51.9)	119 (56.1)	
SINGLE	141 (22.2)	43 (20.4)	46 (21.7)	52 (24.5)	
WIDOWED	97 (15.3)	32 (15.2)	38 (17.9)	27 (12.7)	
Vital signs					
Heart rate, Mean ± SD	84.2 ± 14.5	82.7 ± 15.5	83.5 ± 13.7	86.4 ± 13.9	0.021
Systolic pressure, Mean ± SD	121.0 ± 18.4	119.7 ± 17.8	123.2 ± 19.7	120.0 ± 17.5	0.101
Diastolic pressure, Mean ± SD	65.0 ± 12.0	64.4 ± 11.8	64.6 ± 11.8	65.8 ± 12.5	0.453
Respiratory rate, Mean ± SD	20.1 ± 3.7	19.6 ± 3.8	20.2 ± 3.4	20.5 ± 4.0	0.048
Spo2, Mean ± SD	96.7 ± 2.0	96.8 ± 1.9	96.8 ± 1.8	96.4 ± 2.1	0.056
Temperature, Mean ± SD	36.7 ± 2.4	36.8 ± 0.8	36.6 ± 2.4	36.6 ± 3.3	0.797
Comorbidities					
Cirrhosis, n (%)					0.798
No	601 (94.6)	198 (93.8)	201 (94.8)	202 (95.3)	
Yes	34 ( 5.4)	13 (6.2)	11 (5.2)	10 (4.7)	
Hyperlipidemia, n (%)					0.112
No	309 (48.7)	92 (43.6)	114 (53.8)	103 (48.6)	
Yes	326 (51.3)	119 (56.4)	98 (46.2)	109 (51.4)	
CKD, n (%)					0.409
No	375 (59.1)	121 (57.3)	121 (57.1)	133 (62.7)	
Yes	260 (40.9)	90 (42.7)	91 (42.9)	79 (37.3)	
COPD, n (%)					0.314
No	554 (87.2)	190 (90)	181 (85.4)	183 (86.3)	
Yes	81 (12.8)	21 (10)	31 (14.6)	29 (13.7)	
Hypertension, n (%)					0.783
No	462 (72.8)	150 (71.1)	155 (73.1)	157 (74.1)	
Yes	173 (27.2)	61 (28.9)	57 (26.9)	55 (25.9)	
Laboratory indicators					
WBC, Mean ± SD	12.3 ± 6.8	11.3 ± 5.5	11.5 ± 5.2	14.1 ± 8.8	< 0.001
RBC, Mean ± SD	3.7 ± 0.8	3.7 ± 0.8	3.7 ± 0.8	3.8 ± 0.8	0.43
Platelet Count, Mean ± SD	221.5 ± 96.3	213.8 ± 95.9	226.3 ± 92.7	224.2 ± 100.2	0.36
Hemoglobin, Mean ± SD	10.8 ± 2.3	10.6 ± 2.3	10.8 ± 2.3	10.9 ± 2.3	0.466
Albumin, Mean ± SD	3.2 ± 0.6	3.2 ± 0.6	3.2 ± 0.5	3.1 ± 0.6	0.089
Sodium, Mean ± SD	138.1 ± 5.3	138.5 ± 4.8	138.0 ± 5.6	137.6 ± 5.3	0.205
Potassium, Mean ± SD	4.4 ± 0.8	4.3 ± 0.8	4.3 ± 0.7	4.4 ± 0.8	0.286
Calcium Total, Mean ± SD	8.5 ± 0.8	8.5 ± 0.7	8.6 ± 0.8	8.4 ± 0.9	0.378
Chloride, Mean ± SD	101.5 ± 6.5	102.3 ± 6.5	101.5 ± 6.7	100.8 ± 6.2	0.049
pCO2, Mean ± SD	44.0 ± 13.3	43.1 ± 12.8	44.4 ± 14.3	44.5 ± 12.7	0.467
pO2, Mean ± SD	117.9 ± 97.2	129.3 ± 108.3	117.5 ± 92.4	107.0 ± 89.1	0.061
Free calcium, Mean ± SD	1.1 ± 0.1	1.1 ± 0.1	1.1 ± 0.1	1.1 ± 0.1	0.411
PT, Mean ± SD	16.6 ± 9.7	17.3 ± 11.6	16.4 ± 9.5	16.2 ± 7.8	0.454
PTT, Mean ± SD	42.2 ± 28.4	41.8 ± 27.6	42.3 ± 27.3	42.6 ± 30.4	0.966
HDL, Mean ± SD	37.7 ± 16.2	42.6 ± 17.2	38.2 ± 15.7	32.5 ± 14.1	< 0.001
LDL, Mean ± SD	76.2 ± 43.9	68.2 ± 36.8	72.7 ± 39.3	87.7 ± 52.0	< 0.001
ALT, Mean ± SD	136.0 ± 628.4	112.2 ± 503.0	87.5 ± 413.7	208.1 ± 869.2	0.113
AST, Mean ± SD	255.0 ± 1496.5	178.3 ± 870.4	170.8 ± 1027.8	415.4 ± 2209.0	0.16
Urea Nitrogen, Mean ± SD	37.4 ± 27.7	37.3 ± 29.3	34.4 ± 22.5	40.5 ± 30.6	0.079
Creatinine, Mean ± SD	2.0 ± 1.9	2.0 ± 1.9	1.9 ± 1.9	2.0 ± 1.8	0.782
LD, Mean ± SD	508.8 ± 1134.5	356.9 ± 252.9	399.4 ± 344.6	769.3 ± 1892.5	< 0.001
CK, Mean ± SD	977.3 ± 4448.7	788.2 ± 4119.1	1238.7 ± 5527.9	904.1 ± 3451.5	0.558
CKMB, Mean ± SD	22.0 ± 54.1	15.5 ± 31.2	27.7 ± 71.6	22.9 ± 51.3	0.067
Medication					
Dopamine, n (%)					0.926
No	593 (93.4)	197 (93.4)	199 (93.9)	197 (92.9)	
Yes	42 ( 6.6)	14 (6.6)	13 (6.1)	15 (7.1)	
Epinephrine, n (%)					0.139
No	594 (93.5)	198 (93.8)	203 (95.8)	193 (91)	
Yes	41 ( 6.5)	13 (6.2)	9 (4.2)	19 (9)	
Dobutamine, n (%)					0.093
No	593 (93.4)	196 (92.9)	204 (96.2)	193 (91)	
Yes	42 ( 6.6)	15 (7.1)	8 (3.8)	19 (9)	
Severity of illness scores at admission					
APSIII, Mean ± SD	50.3 ± 19.7	47.4 ± 17.3	49.9 ± 19.7	53.5 ± 21.5	0.006
SAPS-II, Mean ± SD	39.8 ± 13.6	38.3 ± 12.5	40.1 ± 13.2	41.0 ± 15.0	0.126
SOFA, Mean ± SD	5.4 ± 3.7	4.8 ± 3.1	5.0 ± 3.5	6.4 ± 4.1	< 0.001
Outcome					
length of ICU stay, Mean ± SD	7.0 ± 9.0	6.0 ± 6.9	6.3 ± 9.8	8.7 ± 9.8	0.004

%, weighted proportion.; CKD, chronic kidney disease; COPD, chronic obstructive pulmonary disease;

SOFA, Sequential Organ Failure Assessment; SAPS-II, simplified acute physiology score; APSIII, Acute Physiology III; RBC, red blood cell; WBC, white blood cell count; PT, prothrombin time; PTT, Partial Thromboplastin Time; HDL, high density lipoprotein; LDL, low density lipoprotein; ALT, alanine aminotransferase; AST, Aspartate aminotransferase; LD, lactate dehydrogenase; CK, Creatine Kinase; CKMB, creatine kinase isoenzyme.

a P values of multiple comparisons were corrected by the False Discovery Rate method.

b Q1-Q3: Tierce according to Triglyceride-glucose index.

### Association between TyG index and length of hospital stay

**Table 2** displays results from a multivariable logistic regression analysis exploring the association between TyG index and LHS in patients with HF and diabetes. The study investigates β and corresponding 95% confidence intervals (CIs) across varying TyG index to length of hospital stay within this patient cohort.

**Table 2:** Multivariable logistic regression to assess the association of TyG index with length of hospital stay in ICU.

	Model 1		Model 2		Model 3		Model 4		Model 5	
Variable	B 95CI	P_value	B 95CI	P_value	B 95CI	P_value	β_95CI	P_value	β_95CI	P_value
TyG index	1.59 (0.68~2.49)	0.001	1.28 (0.36~2.21)	0.007	1.18 (0.26~2.1)	0.013	1.39 (0.43~2.34)	0.005	1.13 (0.18~2.08)	0.02
TyG index Tierce										
Q1(≤8.984)	0(Ref)		0(Ref)		0(Ref)		0(Ref)		0(Ref)	
Q2(8.984-9.625)	0.31 (-1.4~2.01)	0.726	0.22 (-1.48~1.93)	0.796	0.05 (-1.66~1.75)	0.956	1.26 (-0.33~2.85)	0.12	0.88 (-0.68~2.44)	0.271
Q3(≥9.625)	2.68 (0.97~4.39)	0.002	2.11 (0.38~3.84)	0.017	1.83 (0.1~3.56)	0.038	2.55 (0.83~4.27)	0.004	2.04 (0.33~3.74)	0.02
P for tread		0.002		0.018		0.039		0.004		0.02

TyG index enter as a continuous variable per 1 unit increase.

Model 1: No adjustment.

Model 2: Adjusted for demographic variables (gender, age, marital status, body mass index).

Model 3: Adjusted for demographic variables, Vital signs (heart rate, systolic pressure, diastolic pressure, respiratory rate, SpO2, temperature) Comorbidities (cirrhosis, hyperlipidemia, CKD, COPD, hypertension).

Model 4: Adjusted for demographic variables. Vital signs, Comorbidities, Medication (Dopamine, Epinephrine, Dobutamine), Laboratory indicators (WBC, RBC, platelet count, hemoglobin, albumin, sodium, potassium, calcium total, chloride, pCO2, pO2, free calcium, PT, PTT, HDL, LDL, ALT, AST, urea nitrogen, creatinine, LD, CK, CKMB).

Model 5: Adjusted for demographic variables. . Vital signs, Comorbidities, Medication, Laboratory indicators Severity of illness scores at admission (APSIII,SAPS-II,SOFA).

%, weighted proportion.; CKD, chronic kidney disease; COPD, chronic obstructive pulmonary disease;

SOFA, Sequential Organ Failure Assessment; SAPS-II, simplified acute physiology score; APSIII, Acute Physiology III; RBC, red blood cell; WBC, white blood cell count.; PT, prothrombin time; PTT, Partial Thromboplastin Time; HDL, high density lipoprotein; LDL, low density lipoprotein; ALT, alanine aminotransferase; AST, Aspartate aminotransferase; LD, lactate dehydrogenase; CK, Creatine Kinase; CKMB, creatine kinase isoenzyme; CI, confidence interval; OR, odds ratios; Ref, reference.

In an unadjusted model treating TyG index as a continuous variable, each unit increase in TyG index was associated with a 1.59 higher LHS (β = 1.59; 95% CI = 0.68~2.49; p = 0.001). When categorizing TyG index into three groups and comparing them to the reference group (Q1), the βs for LHS in the second group (Q2) and the third group (Q3) were 0.31 (95% CI: -1.4~2.01, p=0.726) and 2.68 (95% CI: 0.97~4.39, p=0.002), respectively. After adjusting for demographic variables (gender, age, marital status, body mass index) in Model 2, the βs for Q2 were 0.22 (95% CI: -1.48~1.93, p=0.796) and for Q3 were 2.11 (95% CI: 0.38~3.84, p=0.017). Model 3, which further adjusted for demographic variables, Vital signs (heart rate, systolic pressure, diastolic pressure, respiratory rate, SpO2, temperature), Comorbidities (cirrhosis, hyperlipidemia, CKD, COPD, hypertension), showed Q2 β = 0.05 (95% CI: -1.66~1.75, p=0.956) and Q3 β = 1.83 (95% CI: 0.1~3.56, p=0.038). Model 4, including adjustments for demographic variables, Vital signs, Comorbidities, Medication(Dopamine, Epinephrine, Dobutamine),Laboratory indicators(WBC, RBC, platelet count, hemoglobin, albumin, sodium, potassium, calcium total, chloride, pCO2, pO2, free calcium, PT, PTT, HDL, LDL, ALT, AST, urea nitrogen, creatinine, LD, CK, CKMB), revealed Q2 β = 1.26 (95% CI: -0.33~2.85, p=0.12) and Q3 β = 2.55 (95% CI: 0.83~4.27, p=0.004). In Model 5, which also considered Severity of illness scores at admission (APSIII, SAPS-II, SOFA), when TyG index was treated as a continuous variable, each unit increase was associated with a 1.13 higher LHS (β = 1.13; 95% CI = 0.18~2.08; p = 0.02). Categorizing TyG index into three groups, in comparison to the reference group (Q1), the βs for LHS in the second group (Q2) and the third group (Q3) were 0.88 (95% CI: -0.68~2.44, p=0.271) and 2.04 (95% CI: 0.33~3.74, p=0.02), respectively. This trend was statistically significant (P for trend = 0.02).

### Dose–response relationships

**Figure 2:**
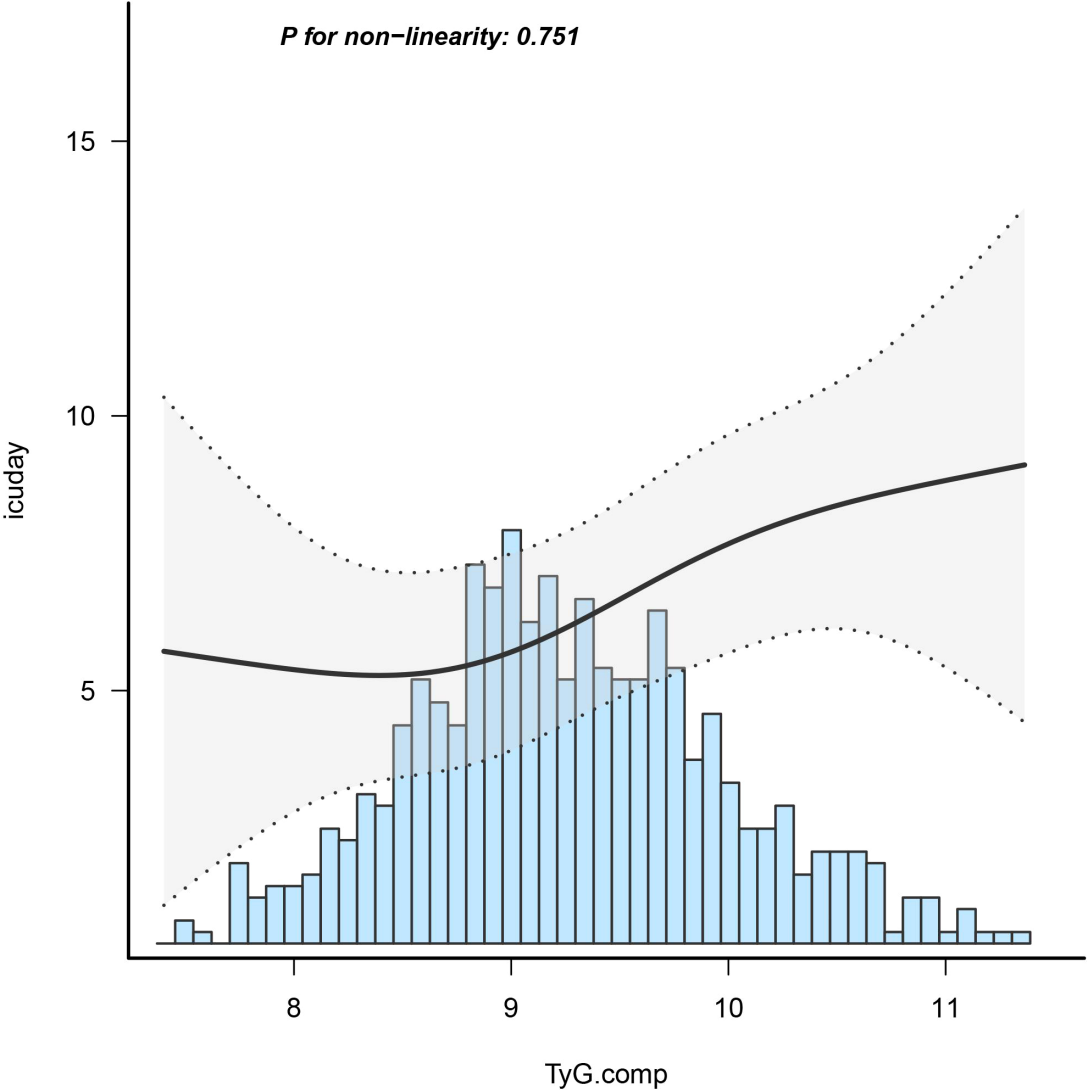
Dose-response relationship between TyG index with length of hospital stay in ICU.

Employing a logistic regression model with a cubic spline function, the study illustrated a statistically significant linear association between TyG index and LHS (p=0.751) after adjusting for confounding factors, as depicted in **Figure 2**.

### Subgroup analysis

**Figure 3:**
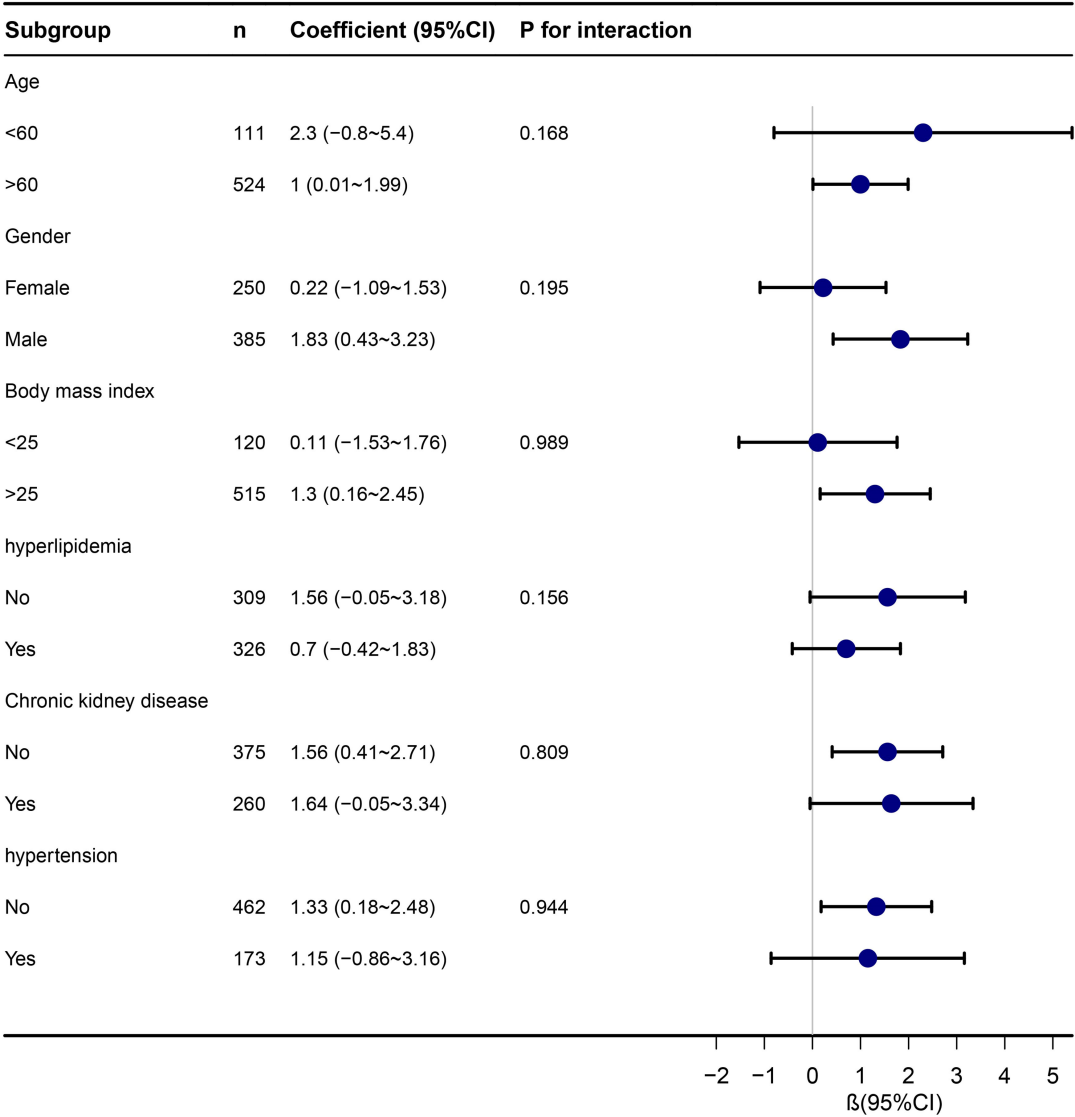
Stratified analyses of the association between TyG index with length of hospital stay in ICU.

Subgroup analyses, stratifying participants by age, gender, BMI, hyperlipidemia, CKD, and hypertension, consistently showed a significant association between TyG index and LHS across various subgroups. The Forrest plot in **Figure 3** highlights the independent and consistent link between TyG index and LHS. Statistically significant associations were not observed in certain subgroups (P > 0.05).

## Discussion

In this retrospective observational cohort study, we present the inaugural investigation into the association between the TyG index and the duration of hospitalization in critically ill patients with coexisting HF and T2D within a United States (US) cohort. Following meticulous adjustment for numerous potential confounding variables, our analysis reveals a positive correlation between the TyG index and the LHS. Employing smooth curve fitting techniques reinforces the identification of a linear relationship between the TyG index and LHS. The robustness of these findings is underscored by stratified and sensitivity analyses.

The TyG index, serving as a novel surrogate marker for insulin resistance, has been extensively utilized in predicting the risk and severity of various diseases (31). Numerous clinical investigations on the association between the TyG index and patient outcomes across diverse diseases have been conducted. Meta-analysis shows that the TyG index as a valuable marker to assess the risk of HF incidence (32), ischemic stroke (33), coronary artery disease (34), cardiovascular diseases and mortality in the general population (35)and atrial fibrillation (36). A study focusing on the general American population demonstrated that an elevated TyG index signifies more severe insulin resistance, displaying a non-linear association with LHS due to all-cause and cardiovascular diseases (37). In the context of chronic kidney disease (CKD), another study found a non-linear relationship between the TyG index and CKD, with a higher TyG index correlating with an increased risk of CKD (38). In the stroke population, a study identified the TyG index as a potential predictor for hospital and intensive care unit (ICU) mortality in critically ill stroke patients (39) Significantly, prior research on the TyG index and its connection to diabetes or HF has primarily focused on individual diseases. A Chinese study revealed an elevated risk of incident T2D with increasing TyG index among rural Chinese individuals, suggesting its potential as an important indicator for identifying individuals at high risk of T2D (40). Another study found that patients with a discordantly high TyG index faced a significantly greater risk of cardiovascular events irrespective of diabetes status (41). Furthermore, an independent association between an elevated TyG index and poor prognosis in patients with acute decompensated HF was identified (17). HF, a prevalent and severe cardiovascular consequence of DM, has garnered substantial global attention (4). Approximately 40% of HF patients are affected by DM, resulting in a two- to five-fold elevation in the risk of HF compared to normoglycemic individuals (42). Previous investigations emphasize the unfavorable clinical prognosis for diabetic patients with HF, displaying higher mortality rates than their normoglycemic counterparts (43–45). Despite the well-established association between diabetes and HF, there is limited data supporting the relationship between the TyG index and the LHS in patients with both HF and T2D. Through multivariate regression analysis and subgroup examinations, our study establishes the independent relevance of the TyG index to the LHS in patients with concurrent T2D and HF. Considering the TyG index as a straightforward method for evaluating insulin resistance, our findings affirm that insulin resistance is independently associated with the LHS.

This study utilized smooth curve fitting and restricted cubic spline analysis to evaluate the linear relationship between the TyG index and the duration of hospitalization in patients with concomitant HF and diabetes, diverging from previous investigations. Subsequent subgroup analyses were conducted to ensure the consistency of the primary findings. The observed association demonstrated a linear pattern, indicating that higher TyG index values are correlated with an increased LHS. These findings carry significant implications for clinical practice and patient care. In our investigation, elevated TyG levels were associated with prolonged in-hospital stays among patients with both T2D and HF within a specific range. This suggests that TyG may serve as a valuable tool for risk stratification and management in this high-risk patient population. Addressing the heightened risk associated with high TyG levels requires a comprehensive approach to risk management. These results underscore the importance of considering the TyG index in clinical practice due to its simplicity, reliability, and convenience, setting it apart from other commonly used methods for disease assessment (46). Researchers have increasingly explored the TyG index, identifying it as a reproducible, reliable, cost-effective, and valid surrogate marker of insulin resistance (IR) (47). Previous studies have demonstrated that the TyG index is highly sensitive (96.5%) and specific (85.0%) for detecting IR compared to the hyperinsulinemic–euglycemic clamp technique (48). Moreover, the TyG index has shown superior performance to Homeostatic model assessment insulin resistance index (HOMA-IR) (49), making it widely applicable in clinical practice, given the availability of glucose and triglyceride tests in all clinical laboratories. Additionally, there may be differences between early onset T2D and late onset T2D, which requires further discussion. One study reviewed the health consequences of early-onset T2DM and compared the results with those of late-onset T2DM, based on matched cohorts in the UK Biobank and reveal that early-onset T2D is more challenging in terms of glycemic control, with a higher risk of severe complications (50). The TyG index may provide an earlier clinical evaluation and provide strategies for future diabetes intervention. Furthermore, from the perspective of medical conditions and promotion, other insulin resistance markers such as HOMA-IR need more structural resources, and access to healthcare. The TyG index can be used as substitute for other tests in clinical settings where these tests are not feasible, and it has been demonstrated that the TyG index has high sensitivity and specificity compared with HOMA-IR and Clamp (48, 51). The TyG index can be easily calculated by TG and FPG without additional cost and has a better performance for the prediction of IR.

Several potential mechanisms elucidate the correlation between the TyG index and LHS. The association with adverse hospitalization duration may be attributed to insulin resistance reducing glucose bioavailability, favoring a shift to fatty acid metabolism, thereby increasing myocardial oxygen consumption and compromising myocardial compensatory capacity (52, 53). Additionally, glycolipid metabolism disorder can induce reactive oxygen species, mitochondrial dysfunction, endoplasmic reticulum stress, impaired cardiac calcium signaling, systemic low-grade inflammation, and inappropriate activation of the renin–angiotensin system, exacerbating heart failure progression (54–56). Furthermore, glycosylation end product deposition may increase diastolic left ventricular stiffness and inactivate nitric oxide, a protective factor for vascular endothelium (57). Lastly, a vicious cycle between congestive heart failure and insulin resistance may intensify cardiac function deterioration (58).

Our study boasts several notable strengths. Firstly, it pioneers the examination of the TyG index’s role in patients with both T2D and HF in the Intensive Care Unit (ICU). Emphasizing the necessity of a comprehensive risk management approach for this patient cohort, our study positions the TyG index as a valuable tool in assessing the LHS. Additionally, we employ restricted cubic splines to explore nonlinear relationships between the TyG index and outcomes. To address potential confounding variables, logistic regression analysis, incorporating multiple models, and subgroup analyses with appropriate categorizations were conducted.

Despite these strengths, our study has acknowledged limitations. Primarily, being a single-center retrospective study limits the definitive establishment of causality. We recommend future validation through multicenter prospective studies. Furthermore, drug use may affect the patient’s condition, but due to database limitations, it may not provide sufficient information on drug use. We plan to investigate the usage of patients more in future research on self-built databases. The baseline-only availability of data impedes the assessment of TyG level changes during the follow-up period, though initial levels likely accurately reflect the TyG index at hospitalization commencement. Then, due to the sample size, there may not be statistically significant differences in some subgroups. However, the overall effect value meets the requirements, and we will explore it in a larger sample size population in the future. Lastly, our findings should not be indiscriminately extrapolated to other countries or ICU facilities, given the study’s confinement to the United States and a single ICU institution. Nevertheless, the considerable sample size enhances the credibility and representativeness of our findings.

## Conclusions

Our study establishes the TyG index as a significant predictor of prognosis in critically ill patients with both HF and T2D, showing an approximately linear relationship with the LHS. In this high-risk group, TyG emerges as a valuable tool for risk categorization and management. To augment validity and coherence, future research should adopt prospective, randomized, controlled study designs to confirm these results and elucidate the mechanisms linking TyG to the LHS in HF and T2D patients.

## Data availability statement

The raw data supporting the conclusions of this article will be made available by the authors, without undue reservation.

## Ethics statement

The establishment of this database was approved by the Massachusetts Institute of Technology (Cambridge, MA, USA) and Beth Israel Deaconess Medical Center (Boston, MA, USA), and informed consents were exempted due to all patients’ data were anonymized before the data were obtained. We also complied with all relevant ethical regulations regarding the use of the data in our study. All reports adhered to the guidelines for Strengthening the Reporting of Observational Studies in Epidemiology and the Declaration of Helsinki.

## Author contributions

KZ: Conceptualization, Writing – original draft. YH: Conceptualization, Writing – original draft. YG: Writing – review & editing, Data curation. FG: Conceptualization, Writing – original draft. TC: Conceptualization, Writing – original draft. ZG: Conceptualization, Investigation, Writing – original draft. ZY: Investigation, Software, Writing – original draft. GM: Conceptualization, Investigation, Writing – original draft. YFG: Conceptualization, Investigation, Writing – original draft. RH: Conceptualization, Investigation, Writing – review & editing. MH: Conceptualization, Investigation, Writing – original draft, Writing – review & editing.
